# PARTICIPATION OF ULTRA-PROCESSED FOODS IN BRAZILIAN SCHOOL CHILDREN’S DIET AND ASSOCIATED FACTORS

**DOI:** 10.1590/1984-0462/2020/38/2019034

**Published:** 2020-06-05

**Authors:** Arabele Teixeira de Lacerda, Ariene Silva do Carmo, Taciana Maia de Sousa, Luana Caroline dos Santos

**Affiliations:** a Universidade Federal de Minas Gerais, Belo Horizonte, MG, Brazil.

**Keywords:** Food consumption, Industrialized foods, Child, Nutritional status, Consumo de alimentos, Alimentos industrializados, Crianças, Estado nutricional

## Abstract

**Objective:**

To evaluate the contribution of ultra-processed foods (UPF) in the schoolchildren diet and associated factors.

**Methods:**

Cross-sectional study with children from public schools in Southeast of Brazil, aged eight to 12 years old. A questionnaire was applied to the students to investigate anthropometric data, eating and lifestyle habits and food consumption. The consumption of UPF was evaluated by two 24-hour dietary recalls (24HR) of non-consecutive days, and the NOVA method was adopted for food classification. The sample was estimated considering the percentage of total caloric value from UPF identified by a similar study (n=260). The association between variables was evaluated by Simple and Multiple Logistic Regression.

**Results:**

The average energy consumption was 1992 kcal/day, 25.2% from UPF. Schoolchildren had a mean of 9.8±0.5 years of age, 53.4% were female and 32.6% were overweight. The most consumed UPF were industrialized pastas, sweet biscuits, sausages, chocolate powder and soft drinks. In the multivariate model, schoolchildren who have the habit of TV watching during meals and those with obesity presented 1.87 (95% confidence interval [95%CI] 1.03-3.39) and 2.05 (95%CI 1,01-4.20) times more chance of having higher consumption of ultra-processed foods, respectively.

**Conclusions:**

The contribution of UPF was expressive in the feeding of the students and it was positively associated with the excess of weight and with the habit of eating while watching television. These findings indicate the importance of nutritional interventions to promote healthy habits, thus preventing overweight during childhood.

## RESUMO

**Objetivo:**

Avaliar a contribuição dos alimentos ultraprocessados (AUP) na dieta de escolares e seus fatores associados.

**Métodos:**

Estudo transversal realizado com crianças entre oito e 12 anos de idade em escolas públicas de uma capital brasileira. Foram investigados dados antropométricos e hábitos alimentares e de estilo de vida. O consumo de AUP foi avaliado por meio de dois recordatórios alimentares de 24 h (R24h) de dias não consecutivos, e o método NOVA foi adotado para classificação dos alimentos. A amostra foi estimada considerando o percentual de valor calórico total proveniente de AUP em estudo semelhante (n=260). Avaliou-se a associação entre as variáveis por meio de modelos de regressão logística simples e múltipla.

**Resultados:**

O consumo médio de energia foi de 1.992 kcal/dia, sendo 25,2% provenientes dos AUP. Os escolares apresentavam média de 9,8±0,5 anos de idade, 53,4% eram do sexo feminino e 32,6% apresentavam excesso de peso. Os AUP mais consumidos foram massas industrializadas, biscoitos doces, embutidos, achocolatado em pó e refrigerantes. Escolares com hábito de comer em frente à televisão e com excesso de peso tiveram 1,87 (intervalo de confiança de 95% [IC95%] 1,03-3,39) e 2,05 (IC95% 1,01-4,20) vezes mais chances de apresentar maior participação de AUP na dieta, respectivamente.

**Conclusões:**

A contribuição dos AUP foi expressiva na alimentação dos escolares e associou-se positivamente com o excesso de peso e o hábito de comer assistindo à televisão. Esses achados denotam a importância de intervenções nutricionais para promoção de hábitos saudáveis, prevenindo assim o excesso de peso na infância.

## INTRODUCTION

In recent years, the high consumption of ultra-processed foods (UPF) has been observed among Brazilian schoolchildren.^1^ Data from the 2015 National School Health Survey (*Pesquisa Nacional de Saúde do Escolar* - PeNSE) show that 41.6, 26.7 and 31.3% of students consume, five or more days of the week, snacks, soft drinks and salty UPF, respectively.^1^ In contrast, only 32.7% of students had adequate consumption of fruits, 37.7% had adequate consumption of vegetables and 60.7% had adequate consumption of beans.^1^

This consumption profile stems from the change in dietary patterns observed in the entire population, which include an increase in the consumption of highly processed products and beverages and the replacement of traditional meals with quick snacks or ready meals, with emphasis on UPFs.^2^

The main purpose of ultra-processing is to create products that are ready for consumption, and this often includes the use of substances such as sugars and fats, which increase the energy density of these foods and make them hyper-palatable.^3^ Thus, UPF have been widely used by families, considering their practicality and children’s high acceptance of them.^2^ ^,^ ^3^ ^,^ ^4^

In Brazil, significant associations between UPF consumption and metabolic syndrome have been observed in adolescents,^5^ with dyslipidemia in children^6^ and with the prevalence of obesity in all age groups.^7^

Given the importance of food consumption not only in terms of nutritional status but also with regard to children’s epidemiological profile, the objective of the present study was to evaluate the inclusion of UPF in the diet of schoolchildren and the associated factors.

## METHOD

This was a cross-sectional study carried out with schoolchildren (between eight and 12 years old) in the fourth year of elementary school in the municipal school system of Belo Horizonte, MG, and with their respective mothers or caregivers. Data were collected between the years 2014 and 2015.

The sample size (n = 260) was calculated using the criteria proposed by Hulley et al.^8^ to estimate proportions, considering the percentage of total caloric value from UPF (21.5%) identified by a similar study,^9^ amplitude of 0.10, significance level of 5% and 80% test power. The values were distributed proportionally to the size of each of the nine regional offices in Belo Horizonte.

Of the schools that had classes of students in the fourth year of elementary school, two teaching units were drawn from each of the regional offices in the city. The selected schools had a total of 931 fourth year students, who were invited to participate in the research. Of these, children who were absent on the day of data collection (n = 101), those who refused to participate in the study (n = 2), or those who had compromised mental health that made their report unfeasible according to the pedagogical teams (n = 31) were not evaluated.

Of the evaluated students (n=797), those whose mother or guardian did not answer the questionnaire were excluded from the study (n=475, response rate of 59.5%). It is worth noting that at least three attempts were made to make telephone contact with the caregivers. They were called three different times of the day. Thus, incorrect telephone number, not answering the call, or technical problems with the number (n = 420) were given as the main reasons for not conducting the interview. In addition, 41 guardians refused to answer the questionnaire via telephone and 14 did not have contact information.

Thus, the final sample was 322 students with their respective mothers or guardians. There was no difference regarding sex, age and nutritional status between the students who were excluded from the study and those who remained in the study (p> 0.05) (data not shown).

Regarding ethics, all mothers or guardians of the children in this study received and signed a Free and Informed Consent Form, and the students’ consent was also obtained. This research was approved by the Research Ethics Committee of the Universidade Federal de Minas Gerais (Presentation Certificate for Ethical Appreciation - CAAE 00734412.0.0000.5149).

For data collection, a face-to-face questionnaire was applied with the students in the teaching units themselves, which included an assessment of food consumption, anthropometry and eating habits and lifestyle. Another was given with their respective mothers or caregivers, by telephone, to investigate socio-demographic and economic variables. The questionnaires used for interviewing the students and their respective guardians were prepared for the study and previously tested. Additionally, the child’s demographic information, such as sex, date of birth and phone number of the guardian, were collected through school documents.

Students’ food consumption was assessed using two 24-hour reminders (R24h), applied on non-consecutive days of the same week, in person. It is worth noting that, at the time of applying the R24h, a list with illustrations of homemade measurements was used, in order to facilitate the identification of the actual portion eaten and provide better consistency of information.

The feasibility of applying R24h to schoolchildren has been previously described.^10^ ^,^ ^11^ The food and beverage consumption data reported by children in homemade measurements were transformed into units of weight (grams) and volume (milliliters) and then associated with the respective information on nutritional composition, according to the methodology proposed by the Brazilian Institute of Geography and Statistics (Instituto Brasileiro de Geografia e Estatística - IBGE)^12^ for processing food consumption data from the Family Budget Survey (*Pesquisa de Orçamentos Familiares* - POF) 2008/2009.

The items present in the food surveys were classified according to the method proposed by Monteiro et al.^3^ (NOVA classification system), which is based on the extent and purpose of food processing. This classification groups all foods and food products into four clearly distinct groups, specifying the type of processing used in their production and the purpose underlying that processing:

- *In natura* food or minimally processed food.
- Processed culinary ingredients.
- Processed food products.
- UPF.^3^

For this study, we considered the UPF group (Chart 1),^3^ by calculating the percentage contribution to total diet energy (% of total caloric value - TCV). This percentage was categorized as less than the 75th percentile and greater than the 75th percentile (75th percentile of the UPF TCV%=33.52%).

**Chart 1:** Specification of the ultra-processed foods evaluated.

Name	Specification
Powdered chocolate	Powdered chocolate
Dairy drinks	Artificial yogurts and boxed and chocolate milk drinks (milk drinks with sugar and chemical additives)
Cookies	Cookies with filling and industrialized sweet cookies
Sweets	Ice cream, chocolates, candies and sweets in general
Sausages	Breaded chicken, sausages, hamburgers and other reconstituted meat products
Instant Noodles	Instant Noodles
Bread products	Sliced bread, hot dogs or hamburgers; sweet breads, cookies, industrialized cakes and cake mixes; breakfast cereals and cereal bars
Sauces and mayonnaise	Tomato sauces, shoyu, ketchup and mayonnaise
Soft drink	Common soft drinks
Salty snakcs	Packaged salty snacks
Artificial juices	Powder for soft drinks/juices

Source: adapted from Monteiro et al.^3^

Among the students, the habit of eating in front of the television and screen time was also investigated through the questions: “do you have the habit of eating in front of the television?” and “How much time do you spend in front of the television, computer or cell phone?”. Daily screen time was categorized as adequate (≤2 hours/day) and inadequate (>2 hours/day), according to the American Academy of Pediatrics.^13^ To assess the habit of consuming school meals, the student was asked whether he or she had eaten one or more meals offered in the teaching units at least three times a week.

In addition, there was an anthropometric assessment of the students, which consisted of measuring weight and height, according to the techniques recommended by the World Health Organization (WHO).^14^ The measurements were performed twice by previously trained health professionals to minimize possible errors in the measurement. Based on the data obtained, the body mass index [BMI = weight (kg)/height(m)^2^] by age was calculated, which was classified according to the criteria proposed by the Food and Nutrition Surveillance System^13^ in view of the WHO growth curves.^15^ Being overweight was considered to be when a participant had BMI-by-age values>Z score+1.^16^

The socio-demographic and economic evaluation included degree of kinship with the child, age, education, marital status and employment status (employed or not) of the mother and/or guardian, family income and number of members in the household. The interviewee’s age was categorized as: <30, 30-59 or ≥60 years. The *per capita* income was calculated as the ratio between all monthly income and the total number of people in the family. Income strata were categorized into up to half a minimum wage or greater than half a minimum wage. The minimum wages in 2014 and 2015 were R$724 and R$788, respectively. Education at home was categorized according to the median number of years of study obtained in this way.

The data collected in the Epi-Info program version 3.4.5 were processed, by typing it out twice, which allowed for a due consistency analysis. Quantitative variables were tested for adherence to normal distribution using the Kolmogorov-Smirnov test. For a descriptive analysis, frequency distributions and measures of central tendency and dispersion were evaluated. To verify the factors associated with the largest UPF consumption quartile, simple and multiple logistic regression models were built. The explanatory variables included in the models were individual characteristics and family context of the students. The multiple model was adjusted for all of these variables. The *Odds Ratio* (OR) with a 95% confidence interval (95% CI) was used as a measure of effect. The data obtained were analyzed using Stata version 12.0 software, adopting a significance value of 5%.

## RESULTS

A total of 322 pairs of students and their mothers and/or guardians were evaluated. The students were an average of 9.8±0.53 years old, 53.4% were female and 32.6% were overweight. As for the children’s guardians, the majority (86.0%) was the mother, with an average of 37.6 ± 9.3 years of age (Table 1). In addition, there was an average of 9.2 ± 2.8 years of education, and 51% had an income *per capita* less than or equal to half the minimum wage.

**Table 1:** Distribution of the sample according to individual and family characteristics of the student.

Variables	n	%
**Children**		
Sex		
Female	172	53.4
Male	150	46.6
Nutritional status		
Not overweight	215	67.4
Overweight	104	32.6
Eats school meal		
No	86	26.9
Yes	234	73.1
Habit of eating in front of the TV		
No	83	25.9
Yes	237	74.1
Screen time		
≤2 hours	171	53.1
>2 hours	151	46.9
**Guardian responsible for care**		
Relationship between guardian and child		
Mother	277	86.0
Father	17	5.3
Others (grandparents, stepmother or aunt)	28	8.7
Age group		
<30 years	52	16.2
30-60 years	259	80.7
≥60 years	10	3.1
Education level		
<9 years of study	133	41.3
≥9 years of study	189	58.7
Per capita income *		
≤½ minimum wage	158	51.0
>½ minimum wage	152	49.0
Marital status		
Married or common-law married	184	57.3
Single, divorced or widowed	137	42.7
Occupation		
Unemployed	141	43.8
Employed	181	56.2

*Minimum wage in 2014 and 2015: R$ 724 and R$ 788, respectively.

The average caloric intake of the students was 1,992.06±951 calories, with 25.2% of the TCV coming from UPF. The most consumed UPF were industrialized bread products, cookies, sausages, powdered chocolate and soft drinks, contributing to 6.35, 2.71, 2.71, 2.39 and 1.95% of TCV, respectively. There was no difference in the consumption of foods evaluated according to the child’s sex (p> 0.05) (Table 2).

**Table 2:** Caloric contribution of the consumption of ultra-processed foods to the total energy consumption of the students.

Foods	Kcal/day/person		Total calories from consumption of ultra-processed food						p-value
			Total	95%CI	Girls	95%CI	Boys	95%CI	
	Average	SD **	%		%		%		
Ultra-processed foods	527.28	24.51	25.22	23.61-26.83	25.15	23.68-26.61	26.39	24.84-27.94	0.481
Bread products	121.89	8.12	6.35	5.55-7.15	6.21	5.11-7.30	6.51	5.32-7.69	0.712
Cookies	123.95	15.34	2.71	2.27-3.16	2.95	2.30-3.60	2.44	1.85-3.04	0.152
Sausages	51.62	4.50	2.71	2.27-3.16	2.95	2.30-3.60	2.44	1.85-3.04	0.258
Powdered chocolate	51.21	4.66	2.39	2.02-2.76	2.47	1.94-3.00	2.30	1.78-2.83	0.670
Soft drinks	41.53	3.82	1.95	1.62-2.28	1.90	1.42-2.38	2.01	1.55-2.47	0.747
Salty snacks	30.48	4.87	1.38	0.95-1.80	1.13	0.60-1.65	1.66	0.97-2.36	0.224
Artificial juice	24.29	1.99	1.28	1.06-1.50	1.32	1.00-1.64	1.23	0.92-1.54	0.689
Sweets	33.82	5.27	1.48	1.08-1.89	1.65	1.04-2.26	1.30	0.76-1.83	0.392
Dairy drinks	21.30	2.78	0.99	0.74-1.25	1.03	0.69-1.37	0.95	0.55-1.34	0.753
Instant Noodles	22.41	5.33	0.98	0.52-1.44	0.97	0.34-1.59	1.00	0.31-1.69	0.938
Sauces	5.78	0.71	0.28	0.21-0.35	0.32	0.22-0.43	0.24	0.15-0.32	0.215

*Student’s *t* test; SD: standard deviation; 95%CI: 95% confidence interval.

In the univariate analysis, the habit of eating in front of the television was a predictor of UPF consumption (OR 2; 95%CI 1.03-3.86). This variable remained in the multivariate model, along with excess weight. Therefore, in the final adjusted model, schoolchildren with the habit of eating in front of the television and who were overweight presented 1.87 (95%CI 1.03-3.39) and 2.05 (95%CI 1.01-4.20) times more likelihood to have greater participation of UPF in the diet, respectively (Table 3).

**Table 3:** Simple and multiple logistic regression analysis between the largest quartile of consumption of ultra-processed foods and individual variables and family context*.

	OR crude (95% CI)	p	Adjusted OR 95%CI	p
Age (years)	1.51 (0.96-2.38)	0.070	1.53 (0.94-2.49)	0.086
Sex				
Female	1.00	-	1.00	-
Male	1.31 (0.78-2.20)	0.283	1.47 (0.84-2.56)	0.177
Nutritional status				
Not overweight	1.00	-	1.00	-
Overweight	1.66 (0.98-2.83)	0.058	1.87 (1.03-3.39)	0.040
Eats school meal				
No	1.00	-	1.00	-
Yes	1.09 (0.60-1.97)	0.769	1.04 (0.54-2.00)	0.886
Habit of eating in front of the TV				
No	1.00	-	1.00	-
Yes	2.00 (1.03-3.86)	0.038	2.05 (1.01-4.20)	0.049
Screen time				
≤2 hours	1.00	-	1.00	-
>2 hours	1.07 (0.64-1.79)	0.790	1.0 (0.60-1.85)	0.846
Variables of the family context				
Age group				
<30 years	1.00	-	1.00	-
30-60 years	1.62 (0.30-8.54)	0.569	1.53 (0.26-9.03)	0.634
≥60 years	1.21 (0.25-5.86)	0.811	0.93 (0.17-4.93)	0.935
Education level				
<9 years of study	1.00	-	1.00	-
≥9 years of study	0.80 (0.48-1.35)	0.412	0.94 (0.52-1.73)	0.865
Per capita income**				
≤½ minimum wage	1.00	-	1.00	-
>½ minimum wage	0.88 (0.52-1.50)	0.654	0.71 (0.39-1.31)	0.284
Marital status (%)				
Married or common-law married	1.00	-	1.00	-
Single, divorced or widowed	1.07 (0.64-1.80)	0.785	0.96 (0.54-1.70)	0.903
Occupation				
Unemployed	1.00	-	1.00	-
Employed	1.62 (0.95-2.76)	0.074	1.60 (0.88-2.90)	0.122

*Dependent variable: 0: less than the 75^th^ percentile of the sample’s ultra-processed consumption (% of total caloric value) and 1: greater than or equal to p75 of the ultra-processed consumption (% of the total caloric value); **minimum wage in 2014 and 2015: R$ 724 and R$ 788, respectively; 95%CI: 95% confidence interval; OR: odds ratio.

## DISCUSSION

The results of the present study showed a high contribution of UPF in the diet of schoolchildren. Additionally, the higher consumption of these foods showed an association with being overweight and the habit of eating in front of the television.

Similar research by Sparrenberger et al.^4^ describes the participation of 47% of UPF in the diet of children in the southern region of the country, a value higher than found here (25.2%). National data presented by the POF (2008/2009) estimate that the UFP contribute on average to 28% of the calories eaten daily.^17^ Even higher participation was observed in the Canadian population, with an average of 61.7% of calories coming from these foods.^18^ Although the value found in this study was lower than that described in the literature, it is emphasized that the consumption of these foods should be avoided, as suggested by the current Food Guide for the Brazilian Population.^19^

Regarding the UPF profile most consumed by the investigated population, industrialized bread products, cookies, sausages, powdered chocolate and soft drinks stand out. Such findings are similar to national data that point out among the most consumed foods, sweets (14.9%), breads (14.1%), snacks and fried foods (10.6%), cookies (5.4%) and soft drinks (3.1%).^20^

The prevalence of being overweight found in this research (32.6%) was similar to that described in a study carried out with children between 6 and 9 years old in the south of the country (34%)^4^. In the Southeast Region, there was a lower prevalence, with 20.6% of children between six and nine years old classified as being overweight.^20^ The increase in the participation of UFP in the diet of the Brazilian population^17^ can be one of the multiple factors that contribute to the growing trend in the prevalence of obesity, especially among children.

In agreement with the national scenario, being overweight among the children evaluated in the present study was associated with a higher intake of UPF. Thus, it is suggested that the excessive consumption of this food group has a detrimental effect on children’s health because it increases the risk of excessive weight gain and other interconnected complications.^21^ Population surveys that have assessed the association between UPF consumption and morbidity and mortality are still scarce due to the recent definition of this food category,^22^ ^,^ ^23^ ^,^ ^24^ however studies already conducted in Brazil indicate significant associations of consumption of these foods with metabolic syndrome in adolescents^5^ with dyslipidemia in children^6^ and with obesity at all ages.^7^

It is believed that this association is at least partially explained by the intrinsic characteristics of these foods, which are hyper-palatable and tend to be consumed in large quantities. In addition, UPF, in general, have high energy density, high levels of total and saturated fats, sugars and sodium, in addition to low fiber content.^25^ This is particularly important when one observes the fact that the consumption of these foods has increased worldwide, in parallel with the global growth of obesity.^16^ ^,^ ^21^ ^,^ ^26^

In the present study, children who habitually ate meals in front of the television were more likely to consume more UPF. The mechanisms that link food consumption in front of the television to a poorer quality diet probably include convenience as a criterion in choosing the type of food to be consumed, which is associated with exposure to food advertisements and changes in the perception of satiety. Foods that are easier to consume are generally more caloric and poor in nutrients. It is already described in the literature that the perception of satiety can be compromised by the distraction caused by television images.^27^

A study carried out by Borzekowski and Robinson^28^ in the United States shows that the display of just 30 seconds of food commercials is already capable of influencing children’s choice to eat a particular product. Additionally, in an analysis of television food advertising on local television channels presented by the study in question, the participation of food and beverages among advertisements was found to be important, and the largest portion (60.7%) of this advertising refers to UPF.^28^

In Brazil, public policies that involve care in the transmission of information through the media are still rudimentary. On the other hand, a significant advancement has been made, with an impact on the food profile of the Brazilian population, that can be assessed in the future. Food processing is now being addressed in the Food Guide for the Brazilian Population.^19^ This version introduces the concept of UPF, enabling it to be identified by the population. In addition, the guide addresses important issues that affect the consumption of these foods, such as supply, cost, time and advertising.^29^

Therefore, it is essential that, in addition to correct information about food, the importance of time dedicated to preparing and consuming meals is also reinforced, in addition to choosing a suitable place to eat. The habit of eating in front of the television gives greater damage to health by favoring the child’s exposure to advertisements for UPF, in addition to affecting the perception of satiety.^30^

Regarding the limitations of this work, socioeconomic homogeneity stands out, as it concerns only students from public schools. Although there is no difference between the students excluded from the sample and those who remained in the study, the low response rate of the guardians should also be considered a limitation, as it was not possible to predict whether there would be differences in the data reported by them. In addition, the cross-sectional design did not allow for the establishment of a causal relationship, only associations between variables. Nevertheless, the research contributes to fill the gap in studies on the consumption of UPF among school-age individuals and brings other strong points, such as the representative sample of children from public schools in a Brazilian capital and the use of a new food classification system.

Therefore, with the results of this work, it was concluded that the contribution of UPF to the diet of the children studied is significant, which shows the poor food consumption of foods that are indicators of a healthy diet. The largest consumption of UPF stands out among children who are in the habit of eating while watching television and who are overweight. In this context, the importance of actions of food and nutrition education is emphasized for the promotion of healthy habits aimed at children and parents, in order to reduce the intake of UPF, mainly in substitution to *in natura* food and minimally processed foods.
